# The Argonaute Proteins ALG-1 and ALG-2 Are Linked to Stress Resistance and Proteostasis

**DOI:** 10.17912/micropub.biology.000457

**Published:** 2021-10-14

**Authors:** Fabian Finger, Franziska Ottens, Thorsten Hoppe

**Affiliations:** 1 Institute for Genetics, University of Cologne, 50674 Cologne, Germany; 2 Cologne Excellence Cluster on Cellular Stress Response in Aging-Associated Diseases (CECAD), University of Cologne, 50931 Cologne, Germany; 3 Novo Nordisk Foundation Center for Basic Metabolic Research, DK-2200 Copenhagen N, Denmark; 4 Center for Molecular Medicine Cologne (CMMC), Faculty of Medicine and University Hospital of Cologne, 50931 Cologne, Germany

## Abstract

The conserved Argonaute-family members ALG-1 and ALG-2 are known to regulate processing and maturation of microRNAs to target mRNAs for degradation or translational inhibition (Bouasker and Simard 2012; Meister 2013). Consequently, depletion of *alg-1* and *alg-2 *results in multiple phenotypes. Our data describe a role of microRNA-regulation in stress resistance and proteostasis with special emphasis on ubiquitin-dependent degradation pathways, such as ubiquitin fusion degradation (UFD) and endoplasmic reticulum (ER)-associated protein degradation (ERAD).

**Figure 1. ALG-1 and ALG-2 Are Linked to Stress Resistance and Proteostasis:**
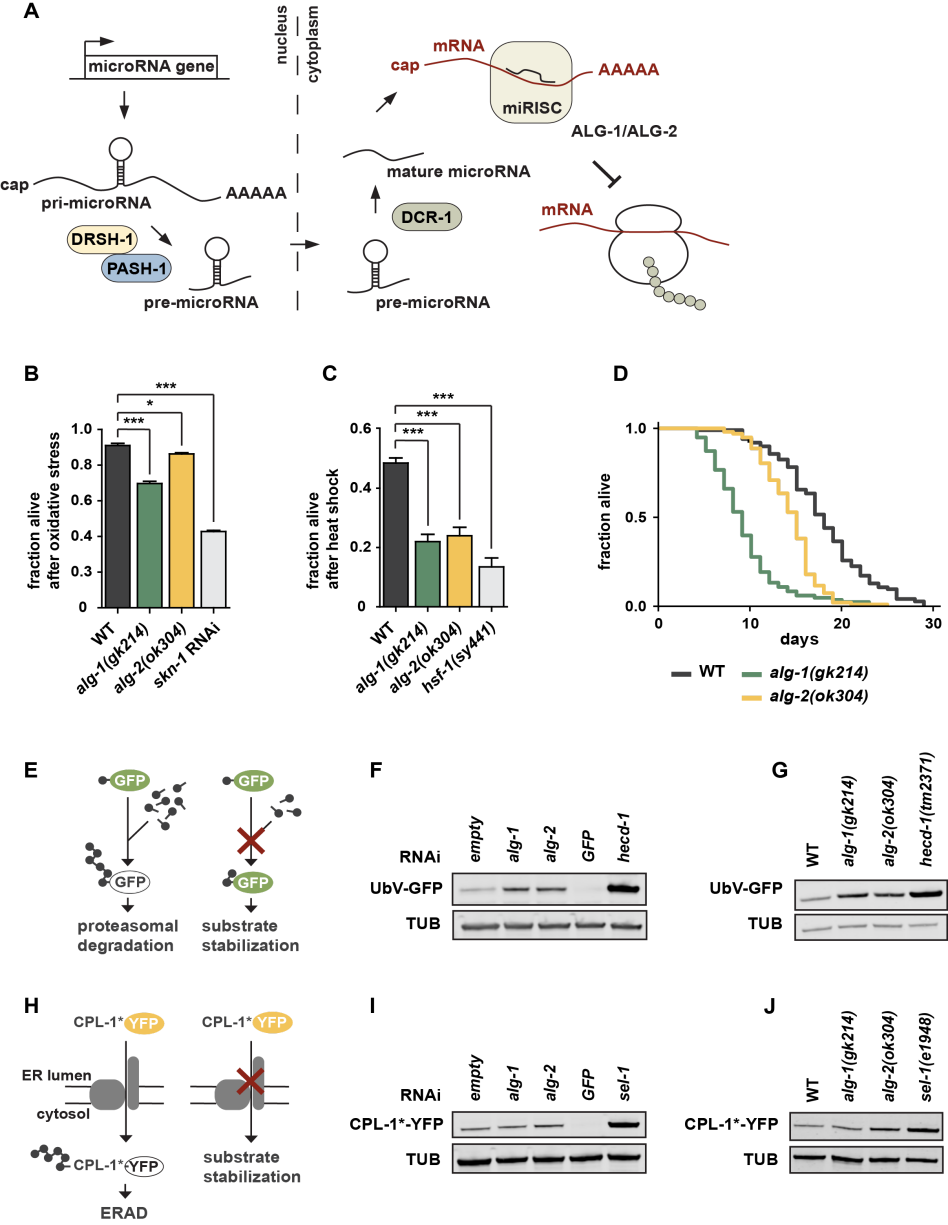
(**A**) Schematic overview on the microRNA pathway in *C. elegans*. (**B**), (**C**) The Argonaute-family members ALG-1 and ALG-2 are required for resistance against oxidative (B) and heat stress (C) compared to wild-type (WT) worms. Data show mean values ± SEM obtained from n = 3 independent experiments using 100-250 worms for paraquat and 50-100 worms for heat stress treatment; *p < 0.05, ***p < 0.001; one-way ANOVA with post-hoc test. (**D**) *alg‑1(gk214)* and *alg-2(ok304)* deletion mutants exhibit short lifespans. Data obtained from n = 2 independent experiments; median lifespan (mean values ± SEM): WT (18.5 ± 0.5 days), *alg-1(gk214)* (9 ± 0.0 days), *alg-2(ok304)* (15 ± 0.0 days). Significance was determined using the Log-rank (Mantel-Cox) test; < 0.0001. (**E**) The ubiquitin fusion degradation (UFD) model substrate monitors ubiquitin-dependent degradation. (**F**, **G**) RNAi-mediated depletion of *alg-1* and *alg-2* (F) and the *alg-1(gk214)* and *alg-2(ok304)* deletion alleles (G) exhibit UFD substrate stabilization. Representative western blots of worm lysates with indicated RNAi and genotypes detecting the UFD substrate (UbV-GFP) and tubulin (TUB). (**H**) The CPL-1*-YFP model substrate monitors ER-associated protein degradation (ERAD). (**I, J**) RNAi-mediated depletion of *alg-2* (I) and the *alg-2(ok304)* deletion allele (J) exhibit increased ERAD substrate level. Representative western blots of worm lysates with indicated RNAi and genotypes showing CPL-1*-YFP and tubulin (TUB) level.

## Description

A fundamental challenge during the life of an organism is to maintain a functional proteome that can adapt to physiological and environmental stresses. Diverse pathways, collectively termed the protein homeostasis (proteostasis) network (PN), provide protein quality control (Roth and Balch 2011; Morimoto and Cuervo 2014). The PN is highly dynamic and continuously adjusted to changing physiological demands. Proteostasis decline results in increased susceptibility to environmental stress and shortened lifespan (Heider *et al.* 2007; Labbadia and Morimoto 2015). Rapid activation of specific stress response pathways, including the heat shock response (HSR), the unfolded protein response in the endoplasmic reticulum (ER) (UPR^ER^) and in mitochondria (UPR^mt^), and the oxidative stress response prevent cellular damage or accumulation of misfolded proteins (Labbadia and Morimoto 2015). However, physiological and environmental changes challenge these stress response mechanisms and trigger increased degradation of damaged proteins. Maintaining the PN depends on changes in gene expression to regulate the protein folding and degradation capacities of the organism. The class of short non-conding microRNAs can bind with partial complementarity to target mRNAs facilitating either mRNA degradation or translational decay (Filipowicz *et al.* 2008). Previous findings showed that microRNAs and the RNAi machinery regulate diverse mechanisms important for development, aging, and stress signaling as well as the development of neurological disorders like myotonic dystrophy type 1 (Alvarez-Garcia and Miska 2005; Smith-Vikos and Slack 2012; Qawasmi *et al.* 2019). We described a role for microRNAs in environmental perception and organismal adaption and identified *mir-71* as a key player in translating sensory inputs from olfactory food cues to the PN in the nematode *Caenorhabditis elegans* (Finger *et al.* 2019). However, the general role of microRNA processing in proteostasis regulation remains unclear.

We studied whether microRNA regulation impacts on proteostasis and stress resistance. We tested whether Argonaute proteins, which are required for microRNA-dependent silencing, are linked to proteostasis in *C. elegans* (Figure 1A) (Tops *et al.* 2006). We characterized the loss-of-function mutants *alg‑1(gk214)* and *alg‑2(ok304),* which lack the two Argonaute-family members important for microRNA function, by using different proteotoxic stress assays (Grishok *et al.* 2001). Although ALG‑1 and ALG‑2 are equally important for processing of most microRNAs, they comprise certain spatiotemporal differences (Tops *et al.* 2006; Vasquez-Rifo *et al.* 2012). Double mutants of *alg-1* and *alg-2* are lethal and were therefore not included here. Both *alg-1(gk214)* and *alg-2(ok304)* mutants showed increased sensitivity towards oxidative stress or heat shock conditions, similar to worms deficient for the transcription factors SKN-1 or HSF-1, which mediate the respective stress response pathways (Figures 1B and 1C) (Hsu 2003; Kahn *et al.* 2008; Park *et al.* 2009). Interestingly, *alg-1* mutants seem to be slightly more sensitive than *alg-2* mutants. Thus, inhibition of microRNA biogenesis and target recognition resulted in increased proteotoxic stress sensitivity, indicating that the respective stress pathways are intimately controlled and maintained by microRNAs. We performed lifespan experiments and detected a reduced lifespan phenotype of *alg-1(gk214)* and *alg‑2(ok304)* worms whereby the *alg-1* mutants showed a more severe reduction compared to *alg-2* (Figure 1D). This is in line with previous findings (Alcedo and Kenyon 2004; Samuelson *et al.* 2007; Ben-Zvi *et al.* 2009; Douglas and Dillin 2010; Kato *et al.* 2011), however, contradicts *alg-2(ok304)*-related lifespan results of the Pasquinelli lab (Aalto *et al.* 2018). Our data support the idea that loss of microRNA biogenesis in adult worms limits stress resistance and reduces lifespan (Lehrbach *et al.* 2012), indicating that microRNA processing and maturation are important for organismal health.

The maintenance of a balanced proteome is supported by ubiquitin-dependent degradation of damaged proteins. We thus used established *in vivo* degradation assays to directly test the involvement of ALG-1/2 in the turnover of an engineered fluorescently labeled model substrates in *C. elegans* (Segref *et al.* 2011, 2014; Denzel *et al.* 2014)*.* The UbV-GFP protein is a short-lived ubiquitin fusion degradation (UFD) substrate, which is expressed under control of the ubiquitous *sur-5* promoter. In wild-type worms, poly-ubiquitylation of the N-terminal ubiquitin moiety triggers degradation of the UFD substrate by the 26S proteasome. Impairment of ubiquitylation or proteasomal turnover result in substrate stabilization, reflected by green fluorescence of the transgenic worms (Figure 1E) (Segref *et al.* 2011). Strikingly, both *alg-1* and *alg-2* deletion mutants affected UFD substrate degradation, similar to *alg-1* and *alg-2* depletion mediated by RNA interference (RNAi) (Figures 1F and 1G). Ablation of the E3 ligase HECD-1, which catalyzes poly-ubiquitylation of the UFD substrate, served as a control exhibiting increased UbV-GFP level. RNAi against *gfp* was used as an additional control to ensure RNAi efficiency. To study whether the defects in UFD turnover reflect a general decline in ubiquitin/proteasome-system (UPS) activity, we also monitored endoplasmic reticulum (ER)-associated protein degradation (ERAD). We followed the turnover of an unstable form of the cathepsin L-like cysteine protease CPL-1 (CPL-1*-YFP), exclusively expressed in intestinal cells by the *nhx-2* promoter (Denzel *et al.* 2014) (Figure 1H). Under standard conditions, CPL-1*-YFP is retro-translocated out of the ER lumen for proteasomal degradation in the cytosol, while loss of the substrate recruiting factor HRD3 homologSEL-1affects ERAD and leads to substrate stabilization (Figure 1I) (Ruggiano *et al.* 2014). Similar to defects in UFD substrate turnover, CPL-1*-YFP levels were also increased in the *alg-2* deletion mutant or upon RNAi depletion (Figures 1I and 1J). In conclusion, our results strongly suggest an intricate link between microRNA processing and ubiquitin-dependent regulation of proteostasis. On this basis, further experiments are warranted to uncover the full range of physiological consequences and mechanistical details.

## Methods

*C. elegans* Maintenance

Nematodes were grown at 20°C (unless stated otherwise) on nematode growth medium (NGM). NGM plates were seeded with the *E. coli* strain OP50 as a food source according to standard procedures and methods (Brenner 1974; Stiernagle 2006). The N2 Bristol strain served as wild-type.

Lifespan and Survival Assays

For lifespan assays 100 age-synchronized L4 larvae per strain were placed to fresh NGM agar plates. Day 0 of the lifespan experiment refers to the first day after reaching adulthood. During the first seven days, animals were transferred on new plates every day to separate from F1s and prevent starvation. After entering the post-reproductive phase, worms were only transferred when necessary. Survival was examined daily by checking avoidance behavior in response to mechanical stimuli or pharyngeal pumping. Experiments were performed in two biological replicates at 20°C. To induce oxidative stress, L4 larvae were incubated in 200 mM paraquat in M9 buffer and survival rates were determined 24 h later, using 100-250 worms in three independent experiments. Heat stress was induced by incubation of age-synchronized L4 larvae at 32.5°C for 15 h. Survival of animals was analyzed in three biological replicates using 50-100 worms each.

RNA Interference (RNAi)

RNAi via the feeding method established for *C. elegans* was performed as described earlier (Timmons and Fire 1998; Kamath *et al.* 2001). Age-synchronized worms were transferred to RNAi plates seeded with *E. coli* HT115 bacteria expressing the respective double-stranded RNA (dsRNA). RNAi clones were either taken from the RNAi Collection (Ahringer) (*alg-1*, *alg-2,* and *hecd-1*) or the ORF-RNAi Resource (Vidal) (*sel-1*) (Rual *et al.* 2004) (Source BioScience). As control condition, bacteria transformed with the empty pPD129.36 vector were used for feeding.

Western Blotting

For whole worm lysates, 100 animals were collected in 80 µl 1x SDS loading buffer. Following, samples were boiled at 95°C for 5 min, sonicated (30 sec at 60% amplitude) and again boiled at 95°C for 5 min. Next, samples were centrifugated at 15,000 rpm for 5 min. For western blotting, protein lysates were resolved by SDS-PAGE using NuPAGE® 4-12% Bis-Tris SDS-gels with the NuPAGE® MES SDS running buffer (ThermoFischer Scientific; settings according to manufacturer’s instructions). Protein transfer was achieved with a semi-dry blotting system (Bio-Rad, Trans-Blot Turbo) with NuPAGE® transfer buffer. Antibodies were diluted in 1x Roti®-Block (Carl Roth). Visualization of fluorescent signals was achieved using the Odyssey scanner (LI-COR) and the Image Studio Lite v4.0 software. All western blots were repeated in n = 3 independent experiments.

## Reagents

List of *C. elegans* strains:

**Table:** 

**Strain**	**Referred to as**	**Genotype**	**Available from**
N2	*WT*	Bristol (N2)	CGC Wormbase ID: N2
*RF54*	*alg-1(gk214)*	*alg-1(gk214)X*	CGC Wormbase ID: WBVar00145621
*WM53*	*alg-2(ok304)*	*alg-2(ok304)II*	CGC Wormbase ID: WBVar00091602
*PS355*	*hsf-1(sy441)*	*hsf-1(sy441)I*	CGC Wormbase ID: WBVar00248994
*PP563*	*UbV-GFP*	*unc-119(ed4)III; hhIs64[unc-119(+); Psur-5::UbV-GFP]III*	Hoppe Lab Segref *et al.*, 2011
*PP1386*	CPL-1*-YFP	*hhIs113[Pnhx-2::cpl-1^W32AW35A^::yfp; Pmyo-2::mCherry]I*	Hoppe Lab
*PP2396*	alg-1(gk214); CPL‑1*‑YFP	*hhIs113[Pnhx-2::cpl-1^W32AW35A^::yfp;Pmyo-2::mCherry]I; alg-1(gk214)X*	Hoppe Lab
*PP2067*	*alg-2(ok304); UbV‑GFP*	*hhIs64[unc-119(+); Psur-5::UbV-GFP]III;alg-2(ok304)II*	Hoppe Lab
*PP2395*	*alg-1(gk214); UbV‑GFP*	*hhIs64[unc-119(+); Psur-5::UbV-GFP]III; alg-1(gk214)X*	Hoppe Lab
*PP1634*	alg-2(ok304); CPL‑1*‑YFP	*hhIs113[Pnhx-2::cpl-1^W32AW35A^::yfp; Pmyo-2::mCherry]I; alg-2(ok304)II*	Hoppe Lab
*PP1536*	sel-1(e1948); CPL‑1*‑YFP	*hhIs111[Pnhx-2::cpl-1^W32AW35A^::gfp]II; sqt-3(sc8) sel-1(e1948)V*	Hoppe Lab
*PP952*	*hecd-1(tm2371); UbV‑GFP*	*hhIs64[unc-119(+); Psur-5::UbV-GFP]III; hecd-1(tm2371)*	Hoppe Lab; Segref *et al.* , 2011

List of antibodies and chemicals:

**Table:** 

**Antibody**	**Source**	**Identifier**
Mouse Monoclonal anti-alpha Tubulin (Clone B-5-1-2)	Sigma-Aldrich	Cat# T6074 RRID:AB_477582
Living Colors® A.v. Monoclonal Antibody (JL-8) (Mouse anti-GFP)	Clontech	Cat# 632380 RRID:AB_10013427
Donkey anti-mouse IRDye® 800CW	LI-COR	Cat# 926-32212 RRID:AB_621847
**Chemical**	**Source**	**Identifier**
Paraquat dichloride hydrate	Sigma-Aldrich	Cat# 36541
